# Halo sign in keratoconus: a case report

**DOI:** 10.1186/s12886-025-04400-5

**Published:** 2025-10-06

**Authors:** Hui He, Lijun Qu

**Affiliations:** Department of Ophthalmology, The Second Affiliated Hospital of Harbin Medical University, Harbin, China

**Keywords:** Keratoconus, Halo sign, Cornea, Optometry

## Abstract

**Background:**

There are several clinical signs that may contribute to the diagnosis of keratoconus. Their appearance is associated with the evolution of keratoconus. In this case, the formation of a halo on the iris in a patient with stage IV keratoconus was reported. This halo sigh is a novel one and has not been reported earlier.

**Case presentation:**

A 30-year-old woman presented to the ophthalmology clinic with one year history of vision deterioration to finger counting in her left eye. A diagnosis of keratoconus was made due to the inferior-central corneal steepening and thinning. When the slit-lamp light projects onto the cone, a halo forms on the iris and shifts its shape and location as the irradiation angle changes.

**Conclusion:**

A halo sign was observed on the iris of a patient with stage IV keratoconus (Amsler-Krumeich classification) when the slit-lamp beam was projected onto the corneal cone.

**Supplementary Information:**

The online version contains supplementary material available at 10.1186/s12886-025-04400-5.

## Introduction

Keratoconus is a corneal ectatic disease with progressive corneal thinning resulting in abnormal corneal shape and astigmatism [1]. Corneal protrusion, corneal thinning, Fleischer’s ring, Vogt’s striae, and Munson’s sign are the prevalent clinical signs of keratoconus [2, 3]. For keratoconus diagnosis, corneal topography and clinical signs provide complementary and indispensable contributions. In the Amsler‒Krumeich staging system, the severity of keratoconus is quantified jointly with the results obtained from slit-lamp examination and corneal topography [4].

## Case presentation

**Fig. 1:**
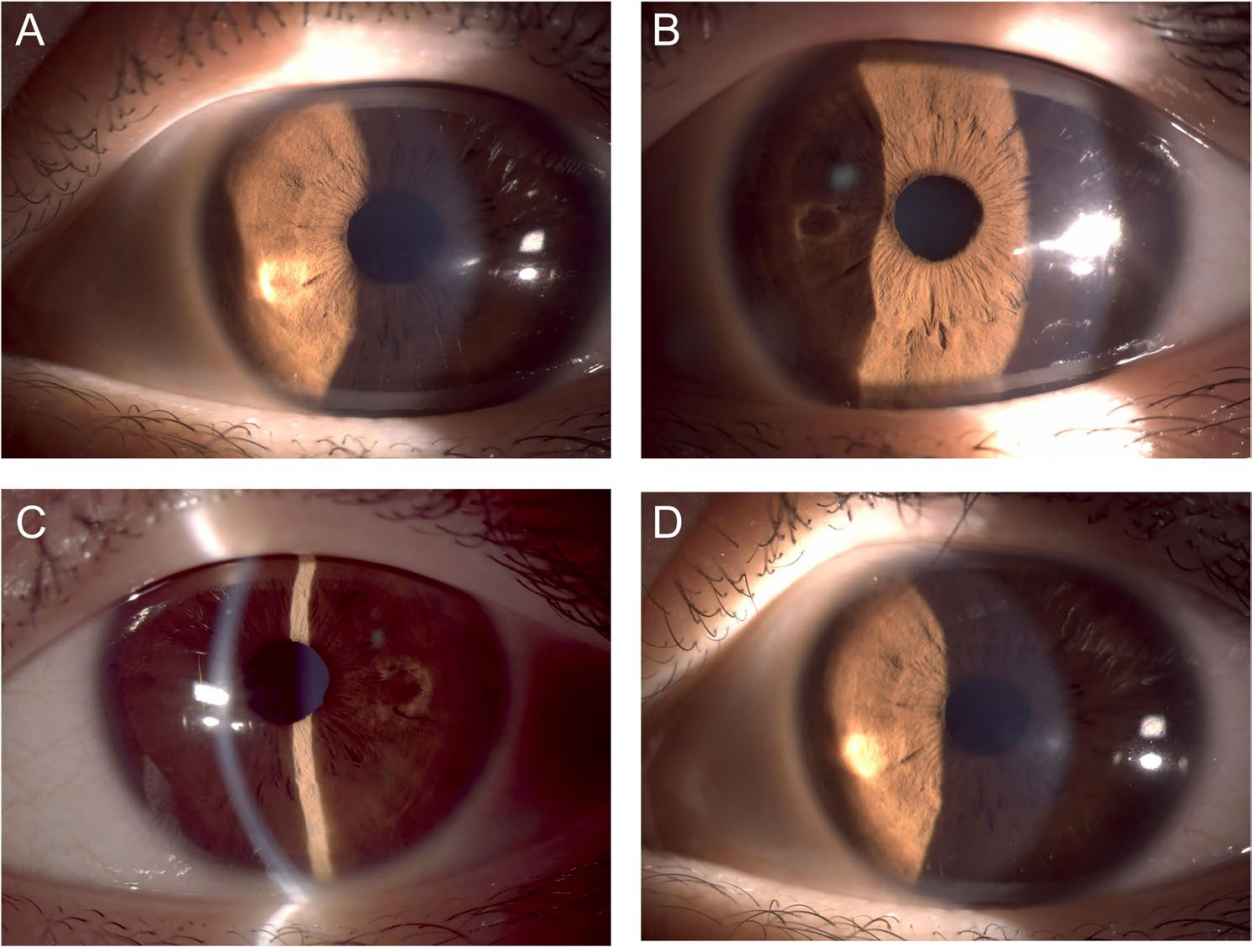
Biomicroscopic appearance of the left eye showing that the halo has different shapes and locations with respect to the irradiation angle of the slit-lamp light. **A** Ring-shaped halo. **B** Oval-shaped halo. **C **Semiring-shaped halo. **D** Dot-shaped halo

A 30-year-old woman presented to the ophthalmology clinic reporting painless visual distortion in her left eye over the past year. Corrected visual acuity measured 20/25 in the right eye but only counting fingers in the left. Slit-lamp examination revealed corneal scarring, Fleischer’s ring, and Vogt’s striae. When slit-lamp light projected onto the cone of the cornea, a halo formed on the iris. The shape and location of the halo changed as the angle of incidence of the beam varied. The shape of the halo was a ring (Fig. 1A), oval ring (Fig. 1B), semiring (Fig. 1C), or dot (Fig. 1D), and it tended to be circular. When the slit beam was located on the temporal side of the eye, the halo formed nasally to the pupil, and vice versa. However, when the slit beam leaves the cone area or irradiates the right eye, a halo on the iris does not form. The halo sign formed only when the light source from the slit-lamp shone on the cone of the left eye, indicating that the optical properties of the cone were altered, leading to the occurrence of the halo.

**Fig. 2:**
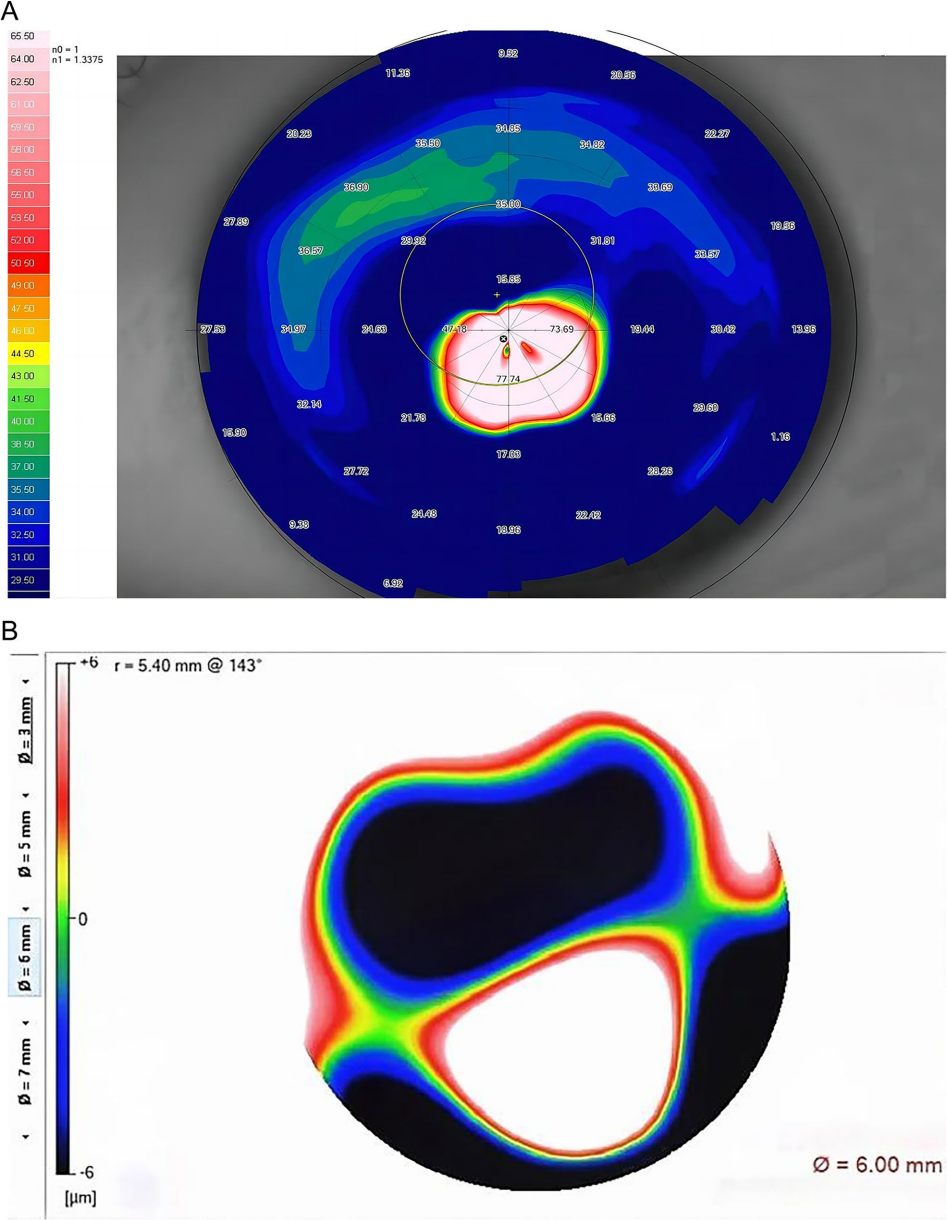
Anterior tangential map(A) and high-order aberration map(B) of the left eye. **A** Nipple cone located in the paracentral cornea. **B** Scale bar corresponds to the magnitude of the aberration. The significantly elevated aberration in the lower region indicates corneal asymmetric deformation, with the cone apex located in the adjacent central area, slightly below the center

Corneal topography revealed that the mean central K value was 59.5 D (Fig. 2A), and the minimum central corneal thickness was 363 μm. The results of these examinations revealed that the left eye was stage IV keratoconus (Amsler-Krumeich classification system). In the assessment of the left eye, a marked elevation in higher-order aberrations was observed (Fig. 2B). Vertical aberration height changes are pronounced in the vertical direction, indicating prominent coma and asymmetric corneal deformation. The anterior protrusion of the cone reduces the depth of field. Eccentric cone rotation generates a trefoil and higher-order astigmatism, creating irregular optical interfaces. These aberrations collectively degrade visual quality. The patient underwent corneal collagen cross-linking (CXL) to inhibit disease progression, with subsequent effective stabilization of the cornea.

## Discussion

Keratoconus is a significant cause of visual impairment in adolescents, so it is important to recognize this condition at presentation to the ophthalmology department. In resource-limited settings lacking access to corneal topography or corneal biomechanical assessment devices, reliance on clinical signs for keratoconus screening becomes particularly critical. Early-stage keratoconus characteristically presents with signs including the oil droplet sign and the scissoring reflex on retinoscopy. As the disease progresses, additional clinical features emerge: the Fleischer’s ring (iron deposition encircling the cone base), Vogt’s striae (vertical stress lines in the deep corneal stroma), and prominent corneal nerves. In advanced cases, Munson’s sign (inferior bulging of the lower eyelid during downward gaze) is typically elicited. Fleischer’s ring and Vogt’s striae are observed in 86% and 65% of affected eyes, respectively [3, 5, 6]. Furthermore, Kriszt [7] proposed that Fleischer’s ring and prominent corneal nerves may serve as diagnostic indicators for subclinical keratoconus.

The halo sign reported in this study is a novel and previously unreported finding. Its underlying optical principles remain incompletely understood. Based on slit-lamp microscopic observations, wave optics theory offers a plausible explanation for its formation. The halo may be caused by an interference pattern resulting from light projection onto the corneal cone. Interference patterns depend on the optical path difference (OPD) between two light beams, which is modulated by the thickness, refractive index, and angle of incidence [8–11]. The cone apex and surrounding cornea create a film of uneven thickness with a concentric distribution, potentially enabling equal thickness interference from reflections and refractions at the anterior and posterior corneal surfaces, manifesting as an annular halo. This mechanism resembles Newton’s ring formed by monochromatic light interference [12]. Notably, the broad spectrum and finite coherence of slit-lamp sources cannot achieve continuous interference fringes as stable as Newton’s rings. Furthermore, factors such as collagen lamellar disorganization and reduced corneal transparency during keratoconus progression may influence optical properties by scattering [13, 14].

This study has several limitations. As a single case report, all observations are derived from one individual. Further studies with larger cohorts and diverse keratoconus subtypes are required to validate the generalizability of the halo sign. The optical principles proposed to explain this sign are based solely on qualitative clinical observations. Interdisciplinary research incorporating optical engineering is needed to clarify its underlying mechanism. Furthermore, the observation of the halo sign is influenced by factors such as the angle of illumination in slit-lamp microscopy, indicating a certain degree of technical dependency.

## Conclusions

The halo sign was observed in a patient with stage IV keratoconus (Amsler-Krumeich classification). Further studies are required to assess its clinical significance.

## Supplementary Information

Supplementary material 1.

## Data Availability

No datasets were generated or analysed during the current study.
