# Supplementary material for: Halo sign in keratoconus: a case report
Source: BMC Ophthalmol. 2025 Oct 6;25:548. doi: 10.1186/s12886-025-04400-5 (PMC12502615; doi:10.1186/s12886-025-04400-5)
Supplement: Supplementary file 1 — Supplementary material 1. [file 12886_2025_4400_MOESM1_ESM.pdf]

Catalogue

Part 1. Slit-lamp photographs of else keratoconus

Figure 1. The other four keratoconus who also had halo sign 1

Part 2. Examination data of the keratoconus who presented in manuscript

Figure 2. Slit-lamp examination with side lighting of the left eye 3
Figure 3. The left eye halo's different morphological – ring 4
Figure 4. The left eye halo's different morphological – oval ring 5
Figure 5. The left eye halo's different morphological – semi-ring 6
Figure 6. The left eye halo's different morphological–pear-shaped ring 7
Figure 7. The left eye halo's different morphological – a dot 8
Figure 8. The halo's different position of the left eye 9
Figure 9. Munson's sign of the left eye 9
Figure 10. Vogt's striae of the left eye 11
Figure 11. Fleischer ring of the left eye 12
Figure 12. Anterior Tangential map of the left Eye 13
Figure 13. Anterior Sagittal map of the left eye 14
Figure 14. Anterior Elevation map of the left eye 15
Figure 15. Posterior Elevation map of the left eye 16
Figure 16. Corneal thickness map of the left eye 17
Figure 17. 3D reconstruction of the left eye anterior surface height 18
Figure 18. 3D reconstruction of the left eye back surface height 18
Figure 19. Anterior segment optical coherence tomography (AS-OCT) of the left eye. 19

Part 3. Machines used

Machines used 21

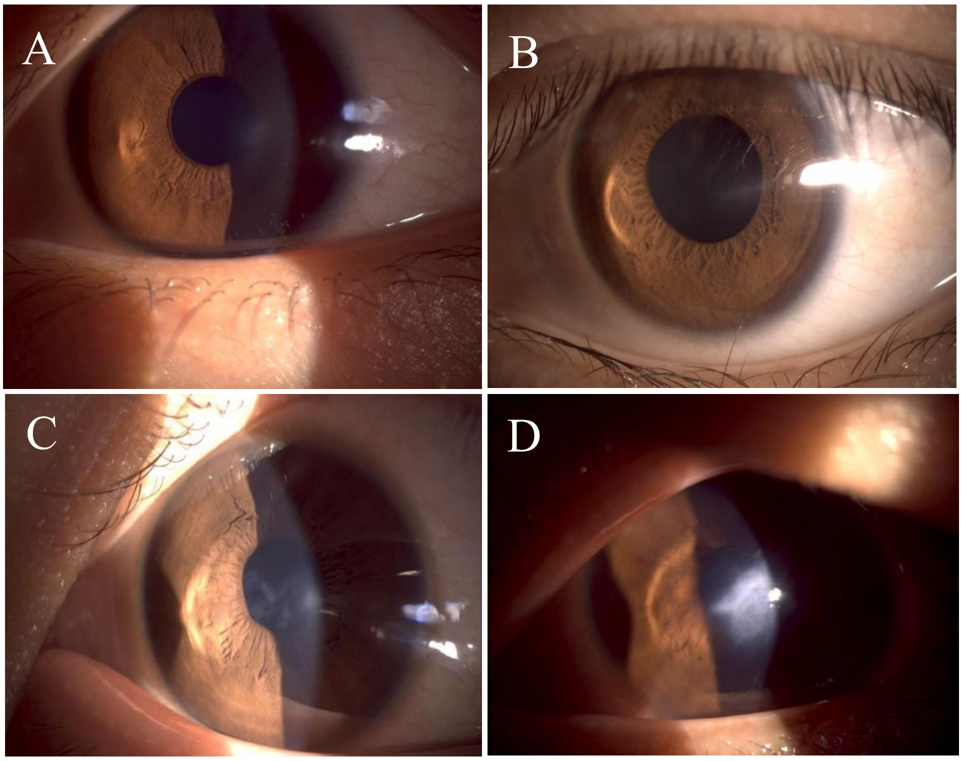

Figure 1. The other four keratoconus who also had halo sign (10×)

Figure1 A. A 24-year-old male with keratoconus in the left front section, where the iris is visible with a complete halo. The affected eye was in keratoconus grade III~ IV.

Figure 1 B. A 27-year-old female with keratoconus in the left front section, where the iris is visible with an incomplete halo. The affected eye was in keratoconus grade IV.

Figure 1 C. A 19-year-old male with keratoconus in the left front section, where the iris is visible with an incomplete and irregular halo. The affected eye was in keratoconus grade IV.

Figure 1 D. A 31-year-old male with keratoconus in the left front section, where the iris is visible with an incomplete and irregular halo. The affected eye was in keratoconus grade IV.

Figure 2. Slit-lamp examination with side lighting of the left eye (10×)

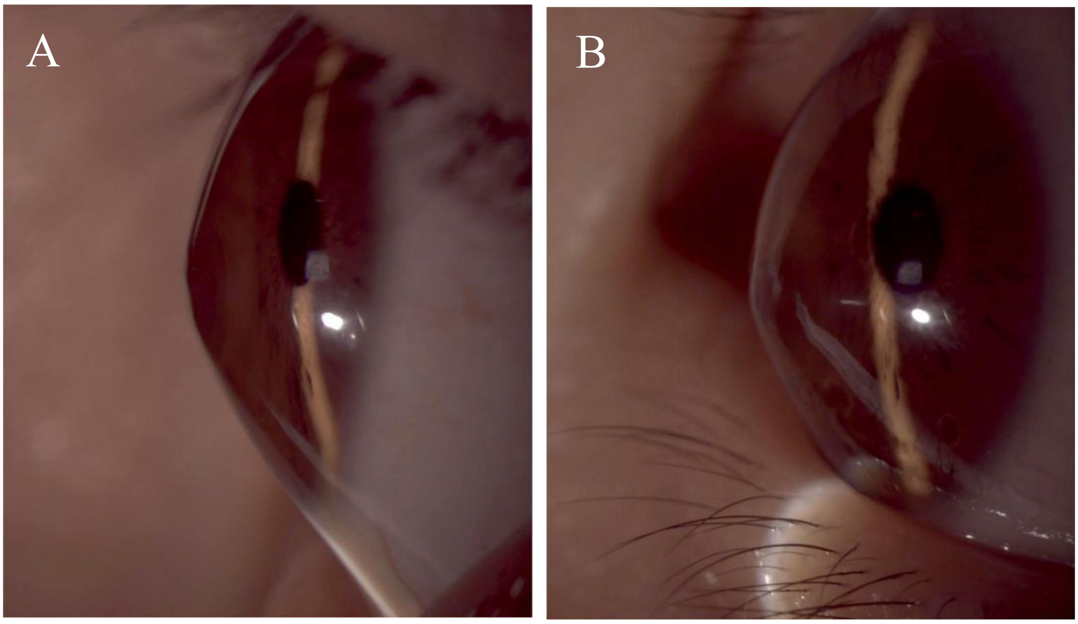

The keratoconus with the cone-shaped corneal protrusion through the Slit-lamp examination with side lighting.

Figure 3. The left eye halo's different morphological – ring (10×)

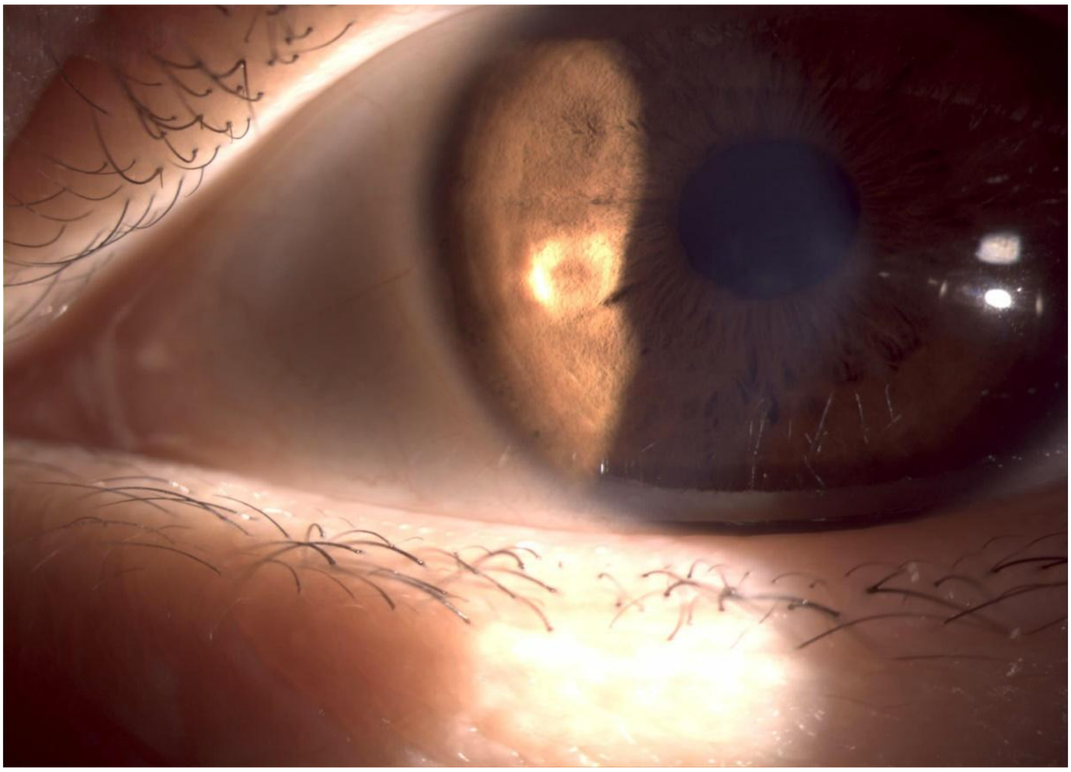

The Slit Beam projected by the Slit-lamp can forms different morphological halos on the iris at different angles. In this figure is a complete ring formed on the iris.

Figure 4. The left eye halo's different morphological – oval ring (10×)

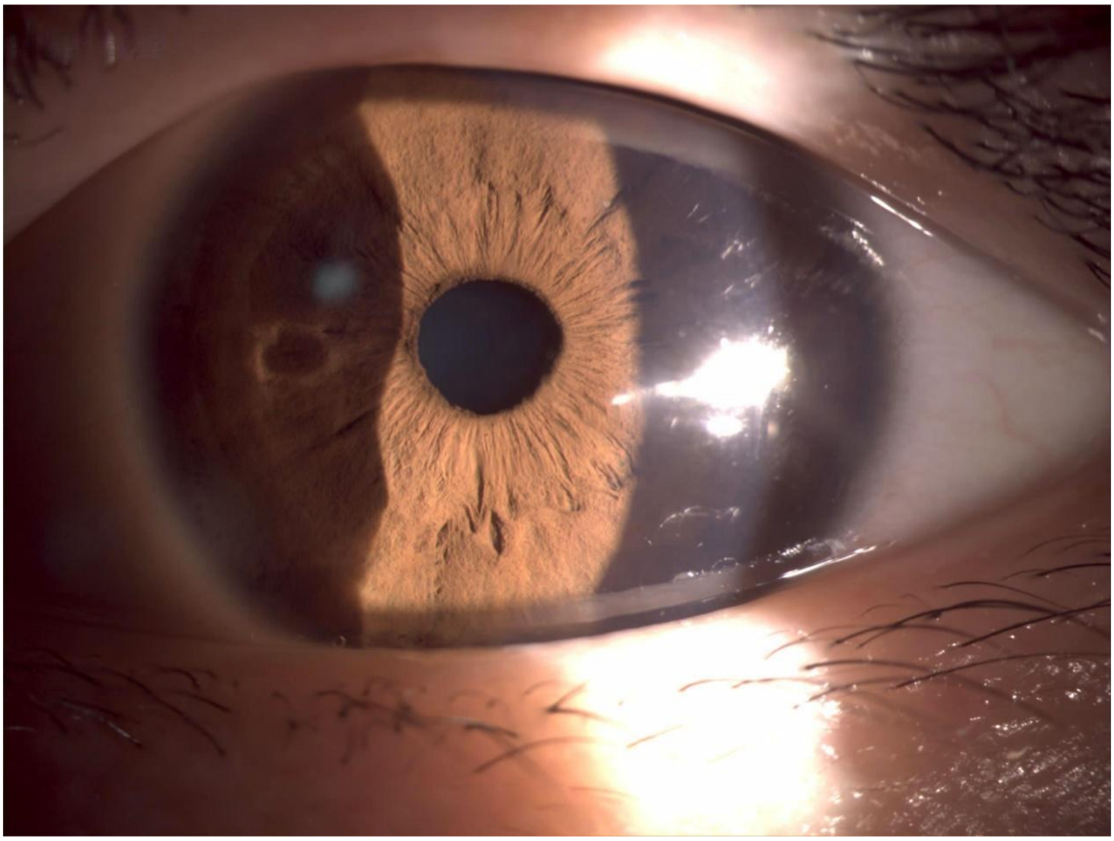

The Slit Beam projected by the Slit-lamp can forms different morphological halos on the iris at different angles. In this figure is an oval formed on the iris.

Figure 5. The left eye halo's different morphological– semi-ring (10×)

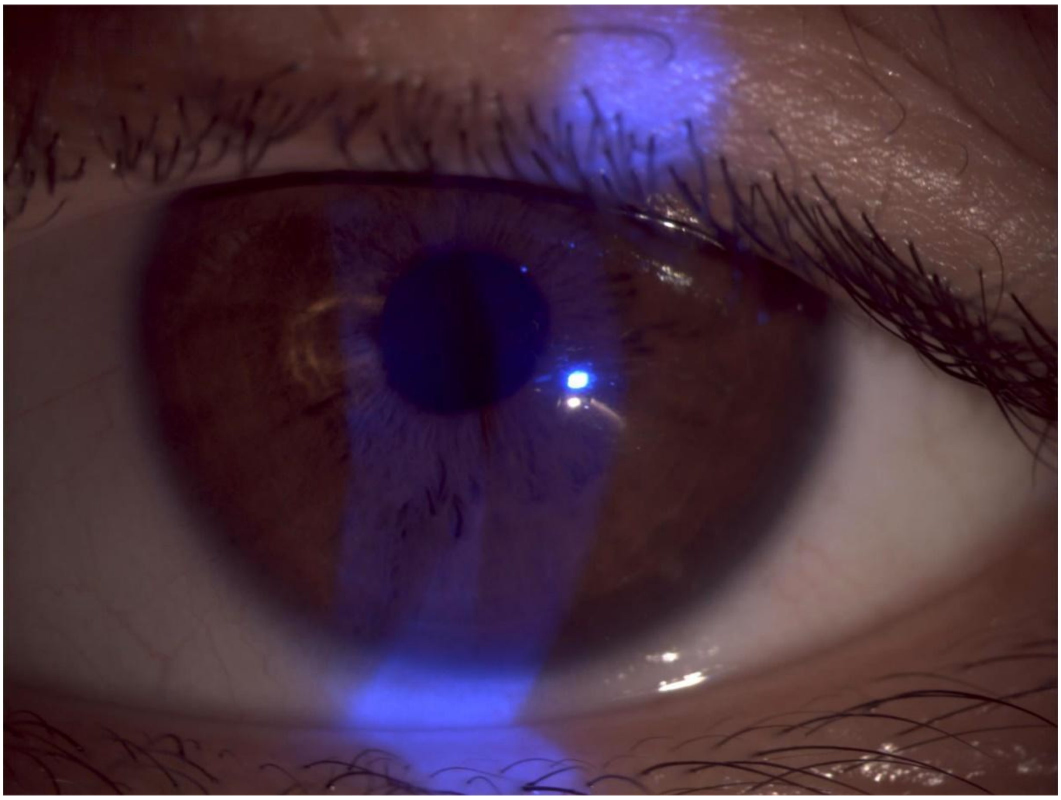

The Slit Beam projected by the Slit-lamp can forms different morphological halos on the iris at different angles. In this figure is a semi-ring formed on the iris (this image was taken under cobalt blue light and background light conditions).

Figure 6. The left eye halo's different morphological – pear-shaped ring (10×)

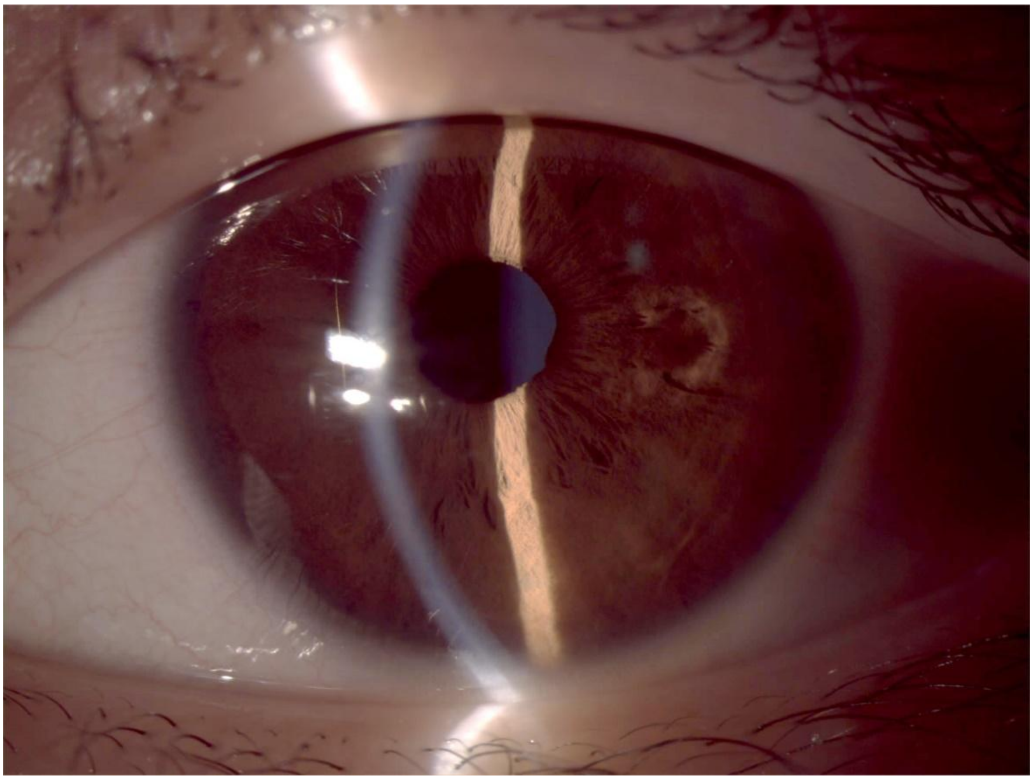

The Slit Beam projected by the Slit-lamp can forms different morphological halos on the iris at different angles. In this figure the halo like a horizontal pear-shaped ring formed on the iris.

Figure 7. The left eye halo's different morphological – a dot (10×)

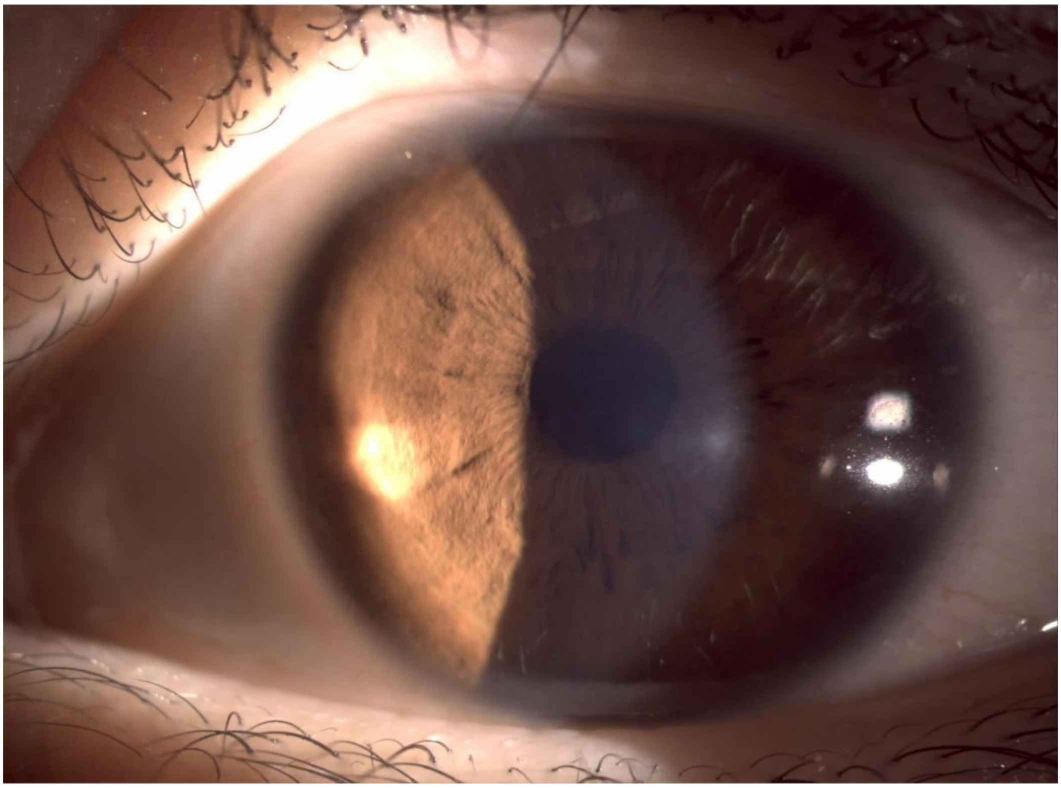

The Slit Beam projected by the Slit-lamp can forms different morphological halos on the iris at different angles. In this figure is a dot formed on the iris.

Figure 8. The halo's different position of the left eye (10×)

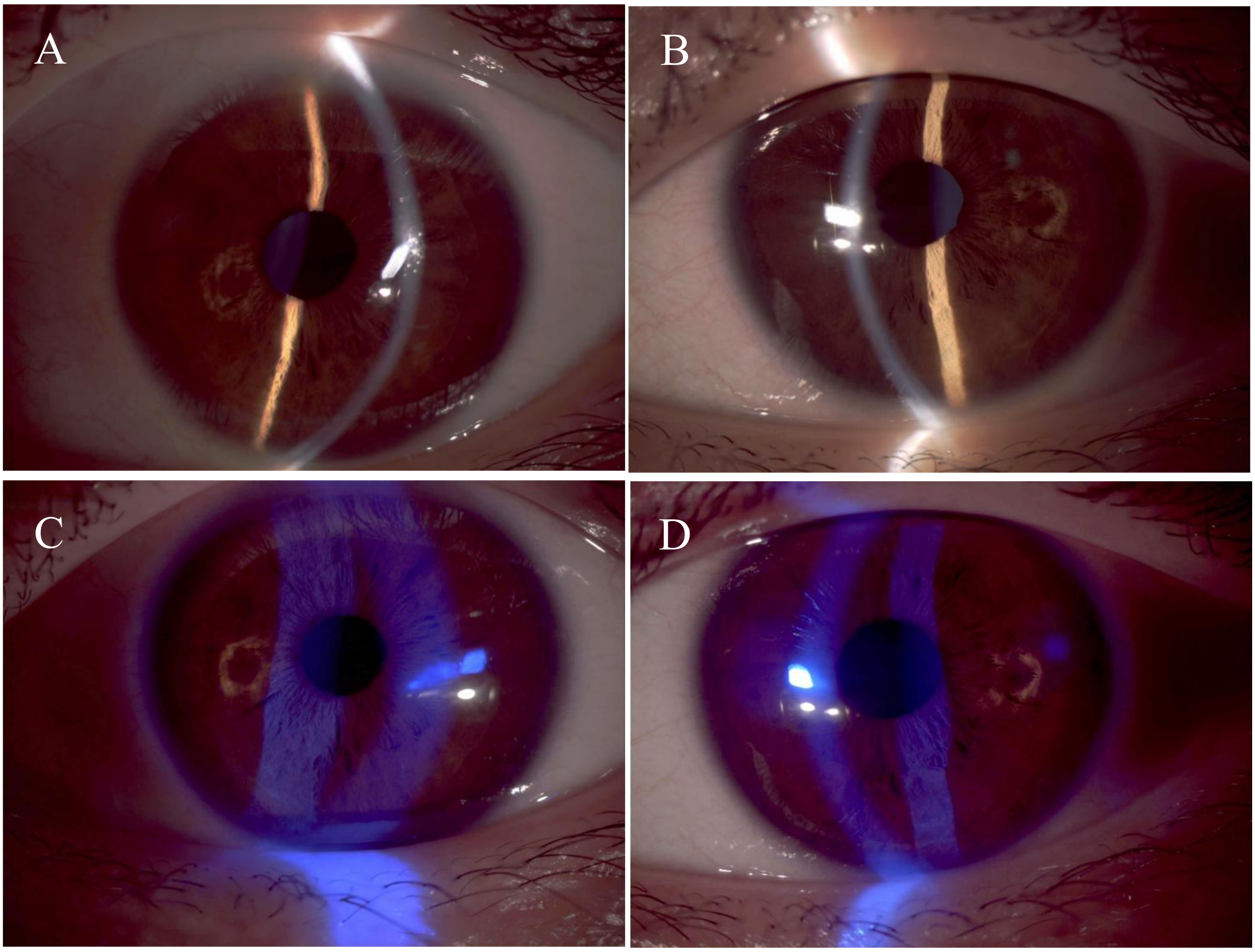

Halos can be formed under both yellow light (Figure 8A and Figure 8B) and cobalt blue light (Figure 8C and Figure 8D), and the position of the halo formation will also change with the angle of projection. When the Slit Beam projected on the nasal side, the halo was formed on the temporal side of the iris (Figure 8B and Figure 8D), and when the Slit Beam projected on the temporal side, the halo was formed on the nasal side (Figure 8A and Figure 8C).

Figure 9. Munson’s sign of the left eye (10×)

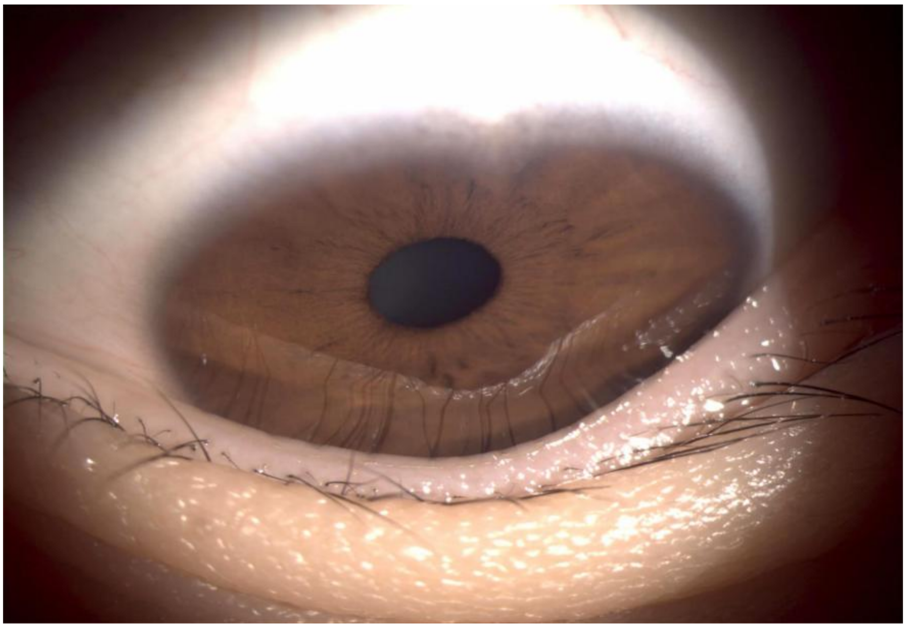

The keratoconus with positive Munson’s sign which is evident as a deformation and protrusion of the lower lid upon down gaze.

Figure 10. Vogt's striae of the left eye

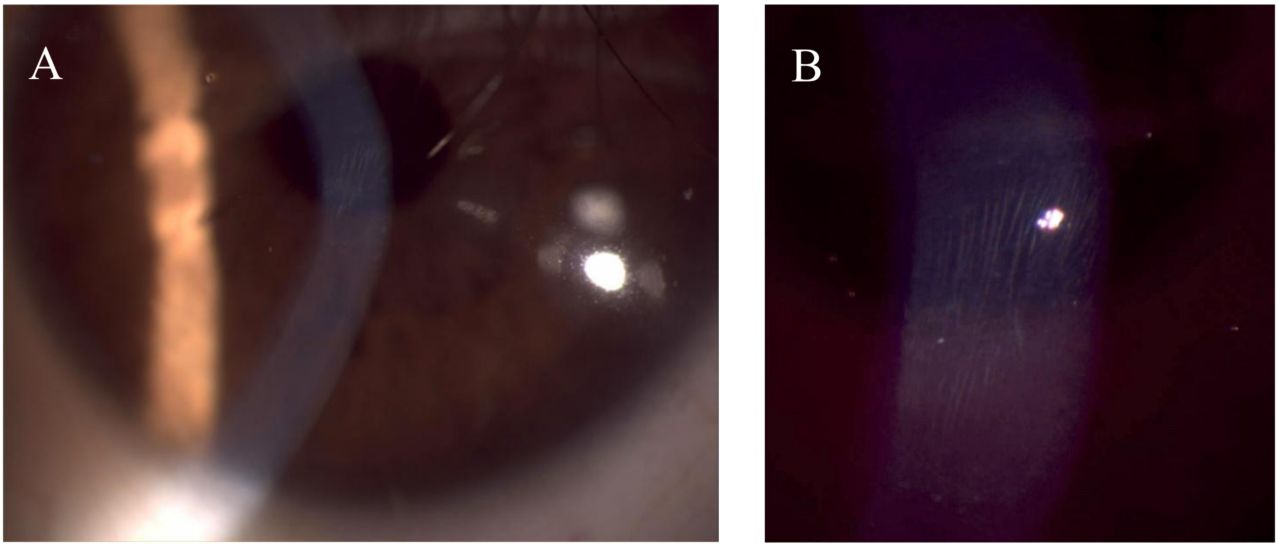

11A. Left Eye (16×)

11B. Left Eye (25×)

The Vogt's striae caused by the corneal stroma stretching and increased folding. The figure shows the vertical Vogt`s striae.

Figure 11. Fleischer ring of the left eye (16×)

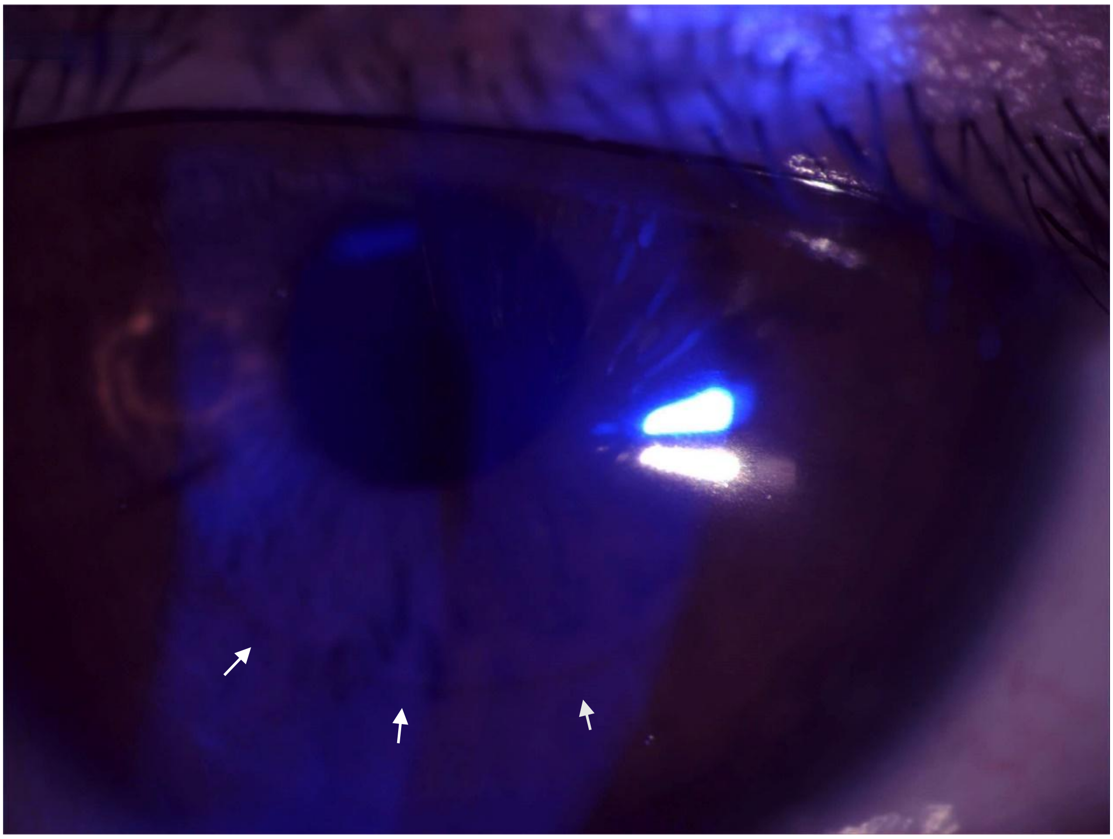

The figure shows the keratoconus with Fleischer ring which is a brown color ring with iron deposits in the corneal stroma of the cone bottom.

Figure 12. Anterior Tangential map of the left Eye

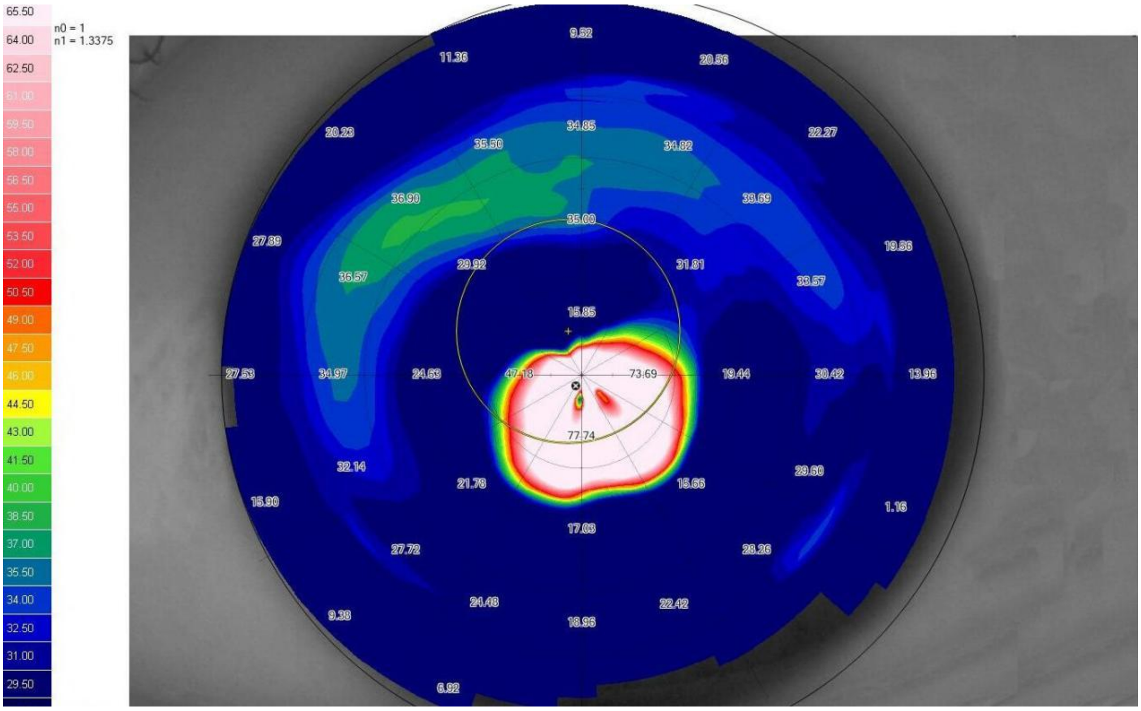

The Anterior Tangential map revealed three changes in corneal curvature from the cone to the peripheral cornea: first, a large decrease around the cone; second, a circular increase in the transition zone; third, a decrease in the remaining corneal tissue.

Figure 13. Anterior Sagittal map of the left eye

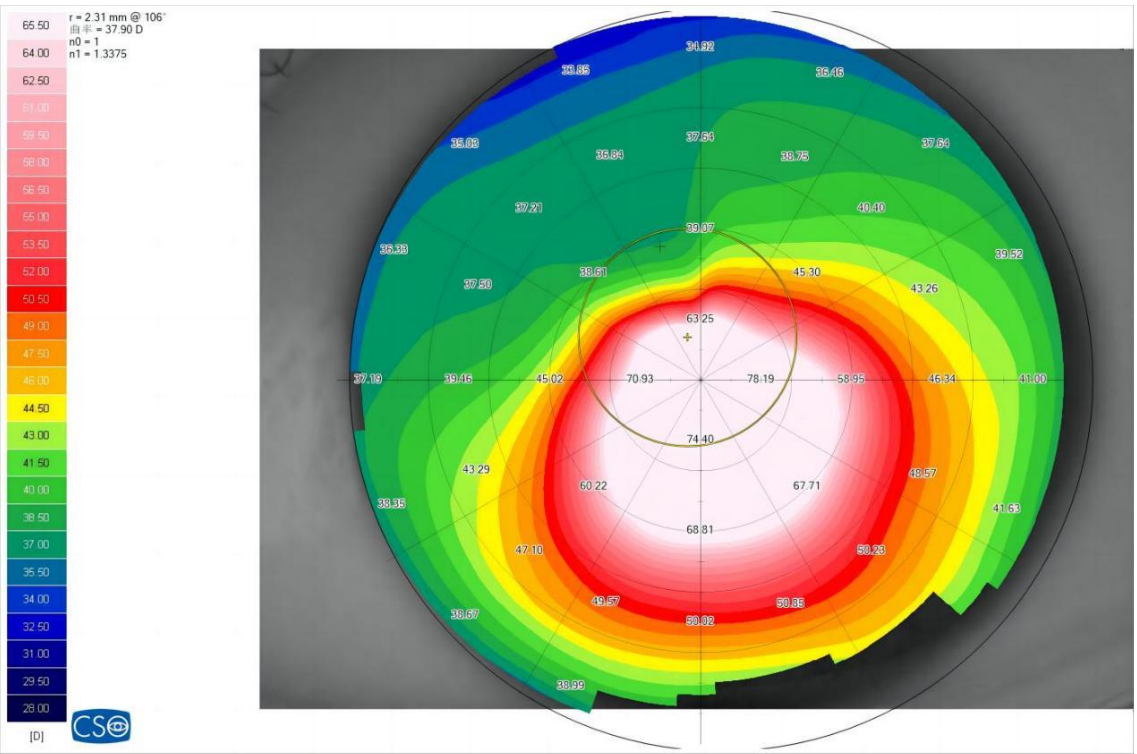

The Anterior Sagittal map shows macroscopic changes in corneal curvature: the area below the cornea is steeper than above.

Figure 14. Anterior Elevation map of the left eye

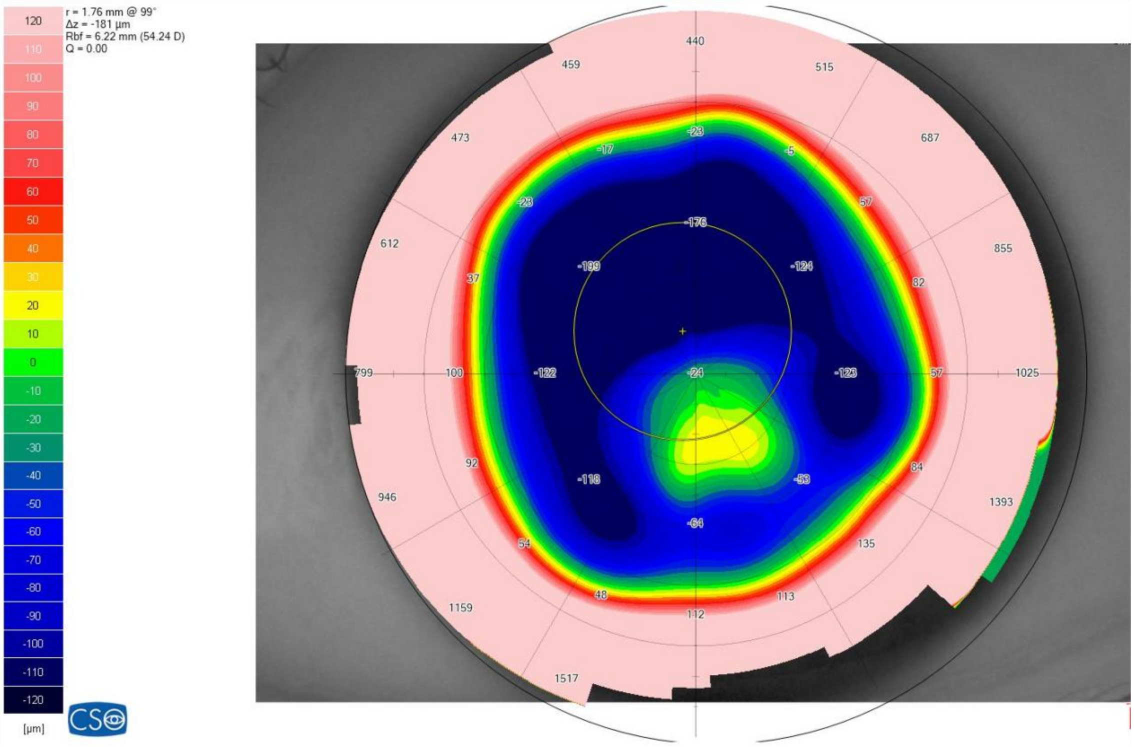

In this figure, the extent of the apex bulge of the cone was not apparent.

Figure 15. Posterior Elevation map of the left eye

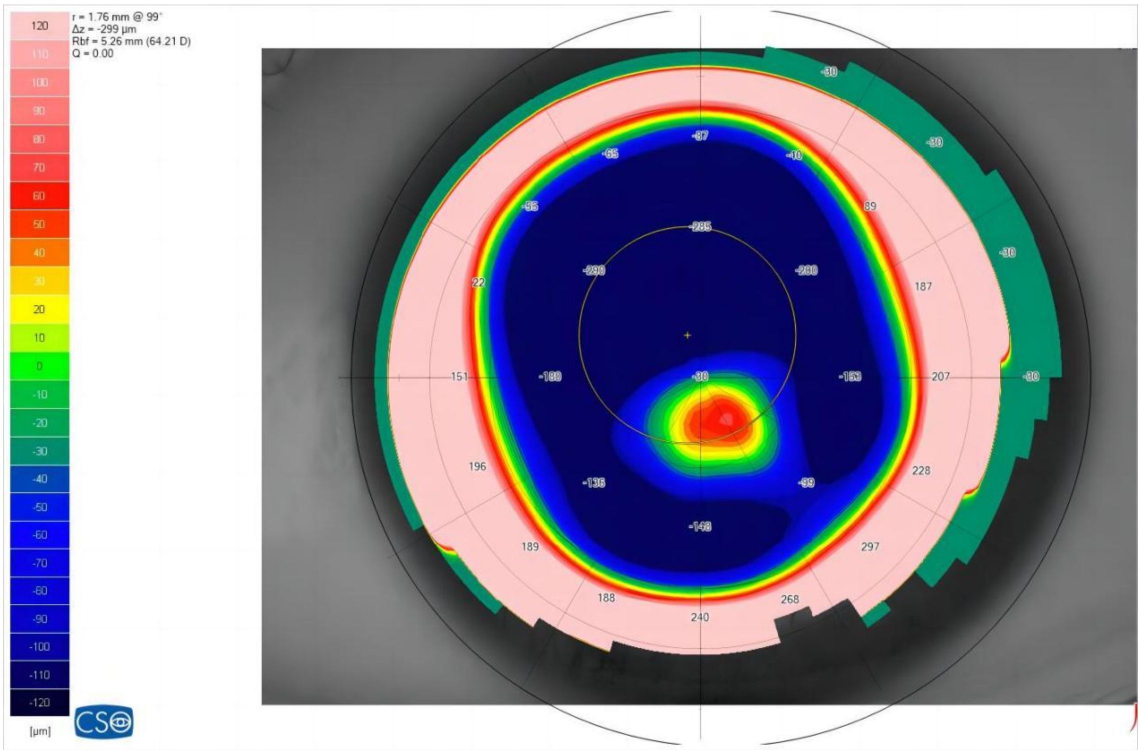

The conical bulge (red) below the posterior surface of the cornea is in marked contrast to the surrounding depression area (blue).

Figure 16. Corneal thickness map of the left eye

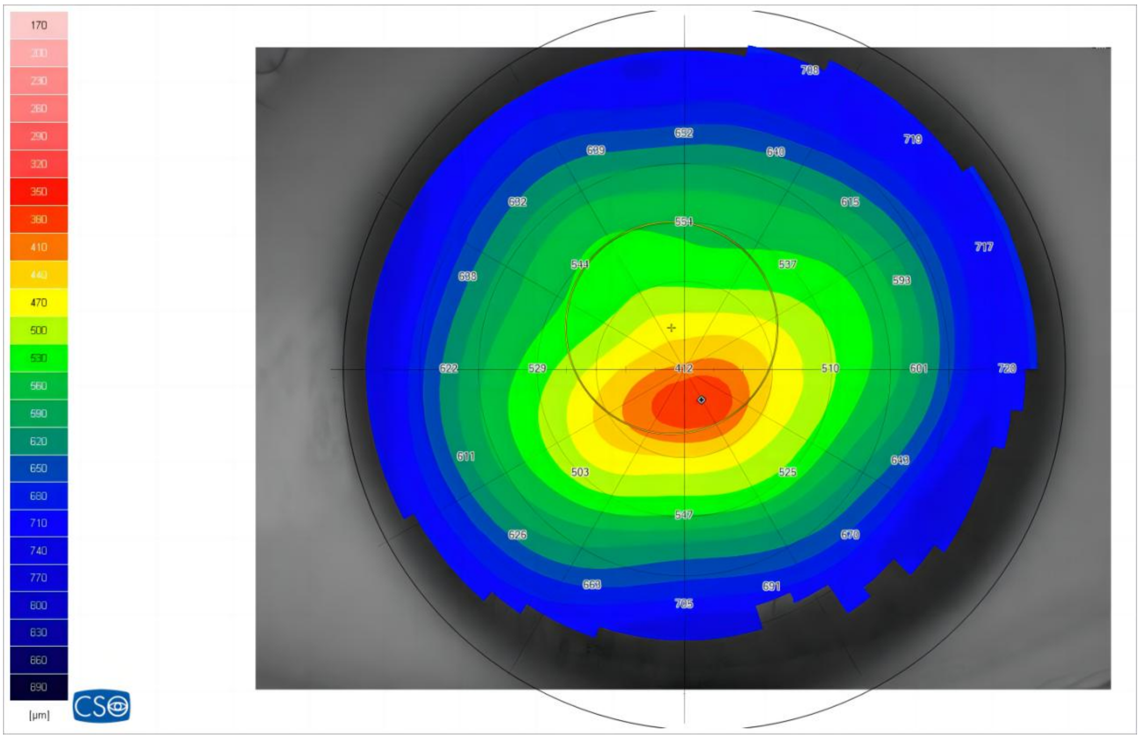

The thinnest point (363μm) of the cornea is below the central cornea. There was a strong trend towards a gradual increase in corneal thickness from the center to the margin.

Figure 17. 3D reconstruction of the left eye anterior surface height

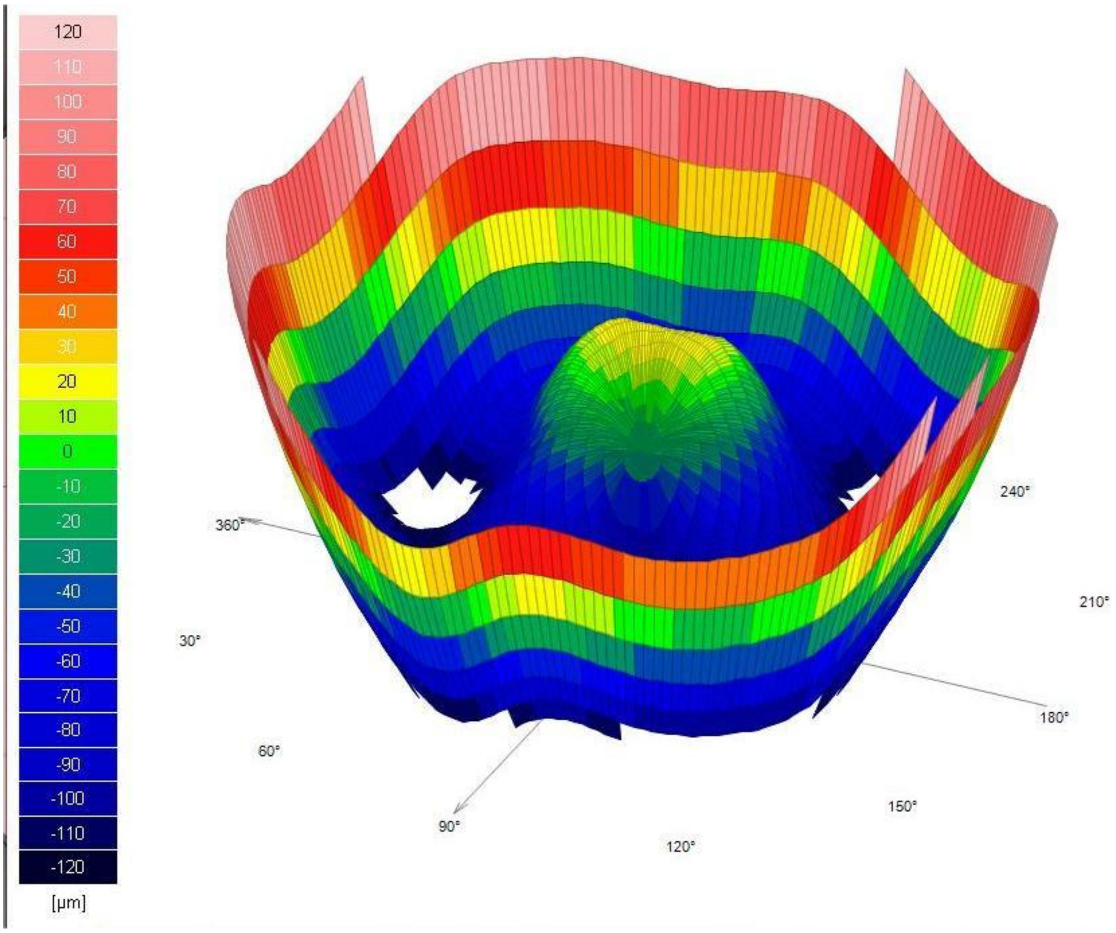

Comparison with the fitted sphere in this case the anterior surface height 3D reconstruction is like a crater pattern.

Figure 18. 3D reconstruction of the left eye back surface height

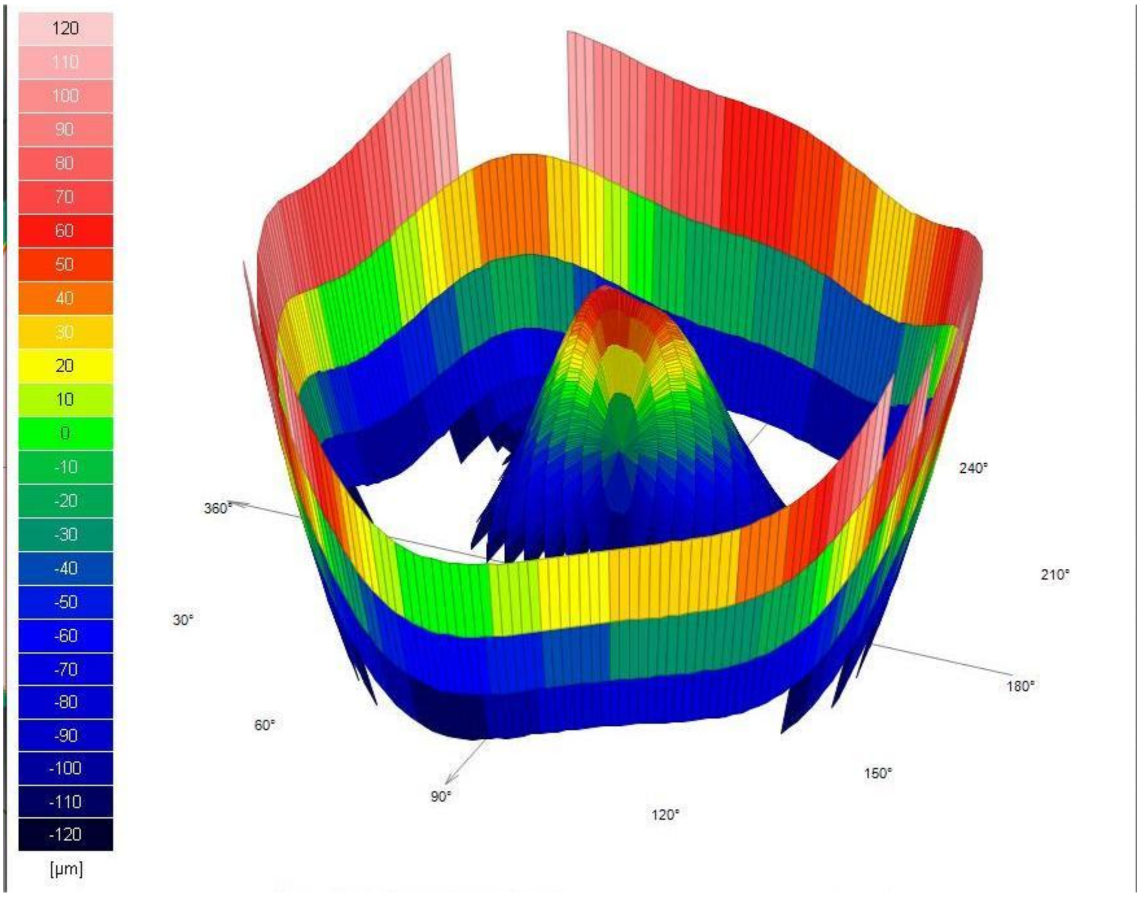

The 3D reconstruction of the back surface height shape is similar to that of the anterior surface height, but the height is higher at the cone.

Figure 19. Anterior segment optical coherence tomography (AS-OCT) of the left eye.

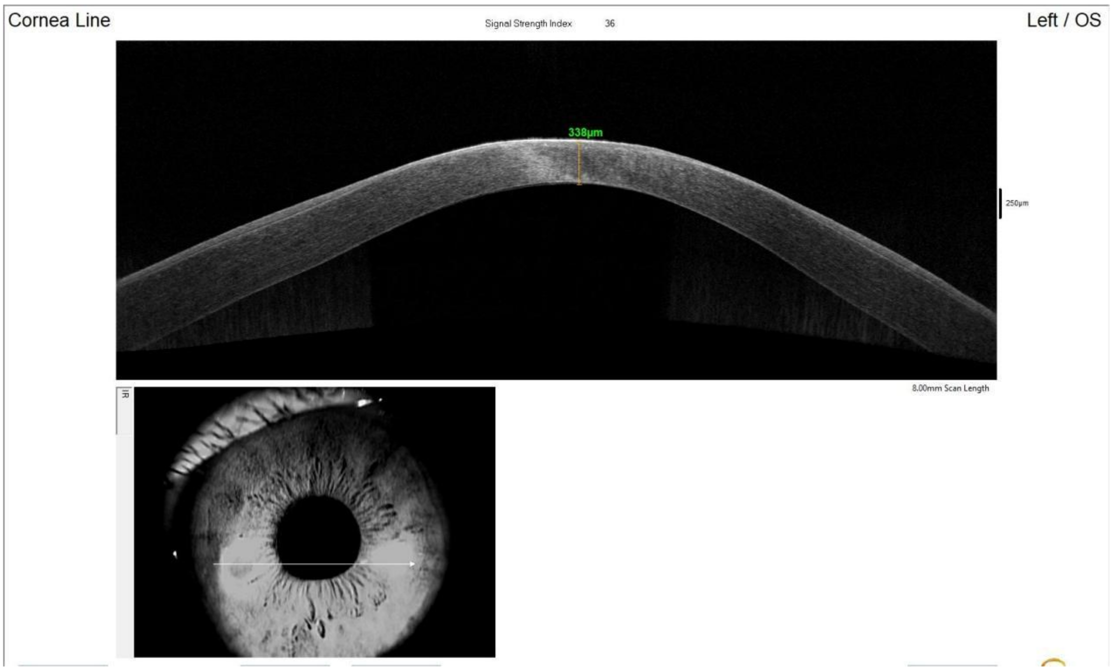

The corneal thickness is heterogeneous, marking thinning in the center, anterior and posterior surface protrusion, and uneven stromal layer.

Machines used

1. **Slit-lamp photograph** from motorized focus digital slit lamp microscope system S390L model, Mediview software.

2. **Corneal topography map** from SIRIUS 3D tomography corneal topography, Phoenix software.

3. **Anterior segment optical coherence tomography angiography (AS-OCTA)** from Ophthalmic Optical Coherence Tomography Angiography System, Avanti Scanner.
